# Supplementary material for: Pyruvate Dehydrogenase Kinase 1 inhibition mediated oxidative phosphorylation enhancement in cartilage promotes osteoarthritis progression
Source: BMC Musculoskelet Disord. 2023 Jul 20;24:597. doi: 10.1186/s12891-023-06585-6 (PMC10357736; doi:10.1186/s12891-023-06585-6)
Supplement: Supplementary file 1 — Additional file 1. [file 12891_2023_6585_MOESM1_ESM.zip › BP.Gsea.1653623667859/GOBP_GLYCOSIDE_METABOLIC_PROCESS.html]

Details for gene set GOBP\_GLYCOSIDE\_METABOLIC\_PROCESS[GSEA]

|  || Dataset | 55457\_collapsed\_to\_symbols.cls.cls #OA\_versus\_normal.cls.cls #OA\_versus\_normal\_repos |
| Phenotype | cls.cls#OA\_versus\_normal\_repos |
| Upregulated in class | OA |
| GeneSet | GOBP\_GLYCOSIDE\_METABOLIC\_PROCESS |
| Enrichment Score (ES) | 0.59363216 |
| Normalized Enrichment Score (NES) | 1.8138556 |
| Nominal p-value | 0.0042735045 |
| FDR q-value | 0.07410178 |
| FWER p-Value | 0.973 |
Table: GSEA Results Summary

  

Fig 1: Enrichment plot: GOBP\_GLYCOSIDE\_METABOLIC\_PROCESS      
 Profile of the Running ES Score & Positions of GeneSet Members on the Rank Ordered List

  

| SYMBOL | TITLE | RANK IN GENE LIST | RANK METRIC SCORE | RUNNING ES | CORE ENRICHMENT || 1 | ABHD10 | "abhydrolase domain containing 10, depalmitoylase [Source:HGNC Symbol;Acc:HGNC:25656]" | 437 | 0.690 | 0.0993 | Yes |
| 2 | AKR1A1 | aldo-keto reductase family 1 member A1 [Source:HGNC Symbol;Acc:HGNC:380] | 808 | 0.567 | 0.1801 | Yes |
| 3 | GUSB | glucuronidase beta [Source:HGNC Symbol;Acc:HGNC:4696] | 904 | 0.541 | 0.2772 | Yes |
| 4 | FUCA1 | alpha-L-fucosidase 1 [Source:HGNC Symbol;Acc:HGNC:4006] | 1506 | 0.428 | 0.3131 | Yes |
| 5 | NAGA | alpha-N-acetylgalactosaminidase [Source:HGNC Symbol;Acc:HGNC:7631] | 1674 | 0.402 | 0.3778 | Yes |
| 6 | GBA3 | glucosylceramidase beta 3 (gene/pseudogene) [Source:HGNC Symbol;Acc:HGNC:19069] | 1767 | 0.388 | 0.4457 | Yes |
| 7 | AKR1C3 | aldo-keto reductase family 1 member C3 [Source:HGNC Symbol;Acc:HGNC:386] | 1996 | 0.361 | 0.4977 | Yes |
| 8 | CBR4 | carbonyl reductase 4 [Source:HGNC Symbol;Acc:HGNC:25891] | 2565 | 0.295 | 0.5106 | Yes |
| 9 | AKR7A2 | aldo-keto reductase family 7 member A2 [Source:HGNC Symbol;Acc:HGNC:389] | 2690 | 0.280 | 0.5551 | Yes |
| 10 | AKR1B1 | aldo-keto reductase family 1 member B [Source:HGNC Symbol;Acc:HGNC:381] | 3138 | 0.236 | 0.5660 | Yes |
| 11 | GBA | glucosylceramidase beta [Source:HGNC Symbol;Acc:HGNC:4177] | 3327 | 0.219 | 0.5936 | Yes |
| 12 | GLA | galactosidase alpha [Source:HGNC Symbol;Acc:HGNC:4296] | 4172 | 0.140 | 0.5552 | No |
| 13 | TH | tyrosine hydroxylase [Source:HGNC Symbol;Acc:HGNC:11782] | 5374 | 0.042 | 0.4701 | No |
| 14 | AKR1C4 | aldo-keto reductase family 1 member C4 [Source:HGNC Symbol;Acc:HGNC:387] | 5843 | 0.005 | 0.4349 | No |
| 15 | AKR1C1 | aldo-keto reductase family 1 member C1 [Source:HGNC Symbol;Acc:HGNC:384] | 8198 | -0.171 | 0.2852 | No |
| 16 | AKR1B10 | aldo-keto reductase family 1 member B10 [Source:HGNC Symbol;Acc:HGNC:382] | 8420 | -0.187 | 0.3041 | No |
| 17 | SLC34A1 | solute carrier family 34 member 1 [Source:HGNC Symbol;Acc:HGNC:11019] | 8919 | -0.226 | 0.3090 | No |
Table: GSEA details [plain text format]

  

Fig 2: GOBP\_GLYCOSIDE\_METABOLIC\_PROCESS      
 Blue-Pink O' Gram in the Space of the Analyzed GeneSet

  

Fig 3: GOBP\_GLYCOSIDE\_METABOLIC\_PROCESS: Random ES distribution      
 Gene set null distribution of ES for **GOBP\_GLYCOSIDE\_METABOLIC\_PROCESS**
